# 

**Published:** 1919-05-24

**Authors:** 


### Spirit Duty (Voluntary Hospitals) Grant.

The Local Government Boards for England and Wales and for Scotland are prepared to receive applications from voluntary hospitals for grants in respect of payments of duty involved by the use during the year 1918, in these hospitals, of duty-paid spirit or drugs containing duty-paid spirits for medical and surgical purposes. Forms of application will be sent only to those hospitals to which a grant was paid last year. Any other hospital which desires to make application should communicate immediately with the Secretary to the Local Government Board for England and Wales, or the Secretary to the Local Government Board for Scotland, as the case may be.

### Passing Events and Topics.

A SITE has been secured at Haltwhistle for building a hospital at an estimated cost of £5,000.

ELLESMERE PORT DISTRICT COUNCIL has appointed a Committee to report on the advisability of building a cottage hospital.

THE collection on the occasion of the annual Infirmary service in St. Chad's Church, Shrewsbury, amounted to £713, as against £500 last year, and not £658 as recorded in THE HOSPITAL.

THE Nottingham Cripples Guild and the Ransom Memorial Trustees have offered to hand over the sum of over £6,000 for the erection of an institution for the treatment of children suffering from surgical tuberculosis.

